# Transfusion requirements after head trauma: a randomized feasibility controlled trial

**DOI:** 10.1186/s13054-018-2273-9

**Published:** 2019-03-12

**Authors:** André L. N. Gobatto, Milena A. Link, Davi J. Solla, Estevão Bassi, Paulo F. Tierno, Wellingson Paiva, Fabio S. Taccone, Luiz M. Malbouisson

**Affiliations:** 1 Internal Medicine, Hospital São Rafael, Salvador, Brazil; 2 Intensive Care Unit, Hospital da Cidade, Salvador, Brazil; 3 Surgical Intensive Care Unit, Anesthesiology Division, Hospital das Clínicas, University of São Paulo Medical School, São Paulo, Brazil; 4 Division of Neurosurgery, Hospital das Clinicas, University of São Paulo Medical School, São Paulo, Brazil; 5 Trauma Intensive Care Unit, Surgery Emergency Department, Hospital das Clínicas, University of São Paulo Medical School, São Paulo, Brazil; 6 Intensive Care Unit, Hospital Alemão Oswaldo Cruz, São Paulo, Brazil; 7 Department of Intensive Care, Erasme Hospital, Brussels, Belgium

**Keywords:** Transfusion, Traumatic brain injury, Anemia

## Abstract

**Background:**

Anemia is frequent among patients with traumatic brain injury (TBI) and is associated with an increased risk of poor outcome. The optimal hemoglobin concentration to trigger red blood cell (RBC) transfusion in patients with TBI is not clearly defined.

**Methods:**

All eligible consecutive adult patients admitted to the intensive care unit (ICU) with moderate or severe TBI were randomized to a “restrictive” (hemoglobin transfusion threshold of 7 g/dL), or a “liberal” (threshold 9 g/dL) transfusion strategy. The transfusion strategy was continued for up to 14 days or until ICU discharge. The primary outcome was the mean difference in hemoglobin between groups. Secondary outcomes included transfusion requirements, intracranial pressure management, cerebral hemodynamics, length of stay, mortality and 6-month neurological outcome.

**Results:**

A total of 44 patients were randomized, 21 patients to the liberal group and 23 to the restrictive group. There were no baseline differences between the groups. The mean hemoglobin concentrations during the 14-day period were 8.4 ± 1.0 and 9.3 ± 1.3 (*p* < 0.01) in the restrictive and liberal groups, respectively. Fewer RBC units were administered in the restrictive than in the liberal group (35 vs. 66, *p* = 0.02). There was negative correlation (*r* = − 0.265, *p* < 0.01) between hemoglobin concentration and middle cerebral artery flow velocity as evaluated by transcranial Doppler ultrasound and the incidence of post-traumatic vasospasm was significantly lower in the liberal strategy group (4/21, 3% vs. 15/23, 65%; *p* < 0.01). Hospital mortality was higher in the restrictive than in the liberal group (7/23 vs. 1/21; *p* = 0.048) and the liberal group tended to have a better neurological status at 6 months (*p* = 0.06).

**Conclusions:**

The trial reached feasibility criteria. The restrictive group had lower hemoglobin concentrations and received fewer RBC transfusions. Hospital mortality was lower and neurological status at 6 months favored the liberal group.

**Trial registration:**

ClinicalTrials.gov, NCT02203292. Registered on 29 July 2014.

**Electronic supplementary material:**

The online version of this article (10.1186/s13054-018-2273-9) contains supplementary material, which is available to authorized users.

## Background

Anemia is common in patients admitted to the intensive care unit (ICU) and is present on admission in 63% of patients. Blood transfusion has traditionally been used to restore hemoglobin concentrations in these patients. Overall, 37% of ICU patients receive at least one blood transfusion during the ICU stay, with a median of five units of packed red blood cells (RBCs) [1]. In patients with traumatic brain injury (TBI), 46% of patients are anemic at some point during their first week of hospital stay and, among those, 76% receive a blood transfusion [2].

Anemia may impair cerebral oxygenation in patients with TBI, especially when hemoglobin concentration is less than 9 g/dL, and blood transfusion may increase cerebral oxygen delivery and potentially reduce the risk of tissue hypoxia [3]. Nevertheless, blood transfusion and anemia are associated with worse outcomes in patients with TBI [2].

Two randomized clinical trials have evaluated different transfusion thresholds in patients with TBI. A post hoc analysis of the Transfusion Requirements In Critical Care (TRICC) study evaluated 67 patients with moderate-to-severe TBI who were included in the original cohort of 838 critically ill patients who had a hemoglobin concentration lower than 9.0 g/dL within 72 h of ICU admission. There were no significant reductions in mortality with a blood transfusion threshold of 10 g/dL compared to a threshold of 7 g/dL [4]. Recently, Robertson et al. conducted a factorial trial to evaluate erythropoietin administration and different hemoglobin thresholds (7 g/dL vs. 10 g/dL) for blood transfusion. Outcomes were not improved with any of the strategies and the transfusion threshold of 10 g/dL was associated with higher incidence of thromboembolic events. Interestingly, anemia was not an inclusion criterion and the patients in both groups had average hemoglobin concentrations greater than 9 g/dL at all reported time points, which may have precluded adequate assessment of the effects of the restrictive transfusion strategy [5].

Transfusion practices in the setting of acute TBI are highly variable, depending on physician specialty and disease severity [6, 7]. We therefore designed a pilot randomized trial to evaluate the feasibility and safety of two different hemoglobin thresholds for blood transfusion in patients with moderate or severe TBI, testing the hypothesis that a larger randomized clinical trial is feasible.

## Methods

### Trial design

Transfusion Requirements After Head Trauma (TRAHT, NCT02203292) was an open-label, parallel, feasibility, randomized controlled trial that was conducted at two ICUs in the Hospital das Clínicas of São Paulo University. The study was conducted in accordance with the amended Declaration of Helsinki. Local institutional review boards approved the protocol (Comissão de Ética para Análise de Projetos de Pesquisa – CAPPesq, reference number 706744) and written informed consent was obtained from all the patients or their surrogates.

### Patients

Patients were considered eligible if they were older than 18 years; were admitted to the ICU with moderate or severe TBI (Glasgow Coma Scale (GCS) score ≤ 12 at hospital admission); and had a hemoglobin concentration less than 9 g/dL within 7 days from hospital admission. Exclusion criteria included patients who had a GCS of 3, with dilated pupils bilaterally; had previous known neurological sequelae; were pregnant; were Jehovah's witnesses; had hemorrhagic shock at randomization; were moribund.

### Study intervention

Enrolled patients were randomized in a 1:1 ratio to the restrictive or the liberal arm in random permuted blocks. None of the investigators or ICU staff members was aware of the randomization list prior to group allocation, or of the block numbers or block sizes, at any time. The randomization was performed using an automated third-party Internet-based service (Sealed Envelope, London, UK) in order to maintain allocation concealment. Given the nature of the intervention, the ICU staff could not be blinded to the treatment assignments. Patients were followed for 6 months after hospital discharge.

Hemoglobin concentrations were assessed at least daily and patients were randomized to the liberal or restrictive blood transfusion strategy when their hemoglobin concentration was less than 9 g/dL. In the liberal group, patients were transfused if the hemoglobin concentration was less than 9 g/dL and in the restrictive group, if their hemoglobin concentration was less than 7 g/dL. Patients were given single units of cross-matched, pre-storage non-leuko-reduced RBCs. After every RBC transfusion, hemoglobin concentrations were checked one hour after transfusion and a single unit of RBCs was provided if the hemoglobin concentrations were lower than 9 g/dL or 7g/dL in the liberal or in the restrictive groups, respectively. The transfusion strategy was respected for 14 days or until death or ICU discharge, whichever came first. All other interventions, including transfusion before ICU admission or during surgery, were at the discretion of the attending team and were not influenced by the trial investigators.

### Outcome measurements

The primary outcome was the difference in mean hemoglobin concentration between the liberal and restrictive groups during the 14 days after hospital admission. Secondary outcomes included the number of transfused patients; the number of RBC packs transfused; ICU mortality; hospital mortality; mortality at 6 months after hospital discharge; adverse events; presence of elevated intracranial pressure (ICP) and intensity of intracranial hypertension treatment [8]; cerebral hemodynamic findings on sequential transcranial Doppler ultrasound (TCD) analysis; lengths of ICU and hospital stay; ICU-free days; duration of mechanical ventilation; mechanical-ventilation-free days and neurological status at hospital discharge and 6 months after hospital discharge. Withdrawal of care was at the discretion of the attending medical team, and was not influenced by the investigators.

A TCD examination was performed every 48 h and mean cerebral arterial velocities, pulsatility indexes and Lindegaard indexes were measured. Post-traumatic vasospasm was defined as a middle cerebral velocity greater than 120 cm/sec in one vascular territory associated with a Lindegaard Index greater than 3 [9, 10].

An adverse event was defined as the occurrence of any of the following: septic shock [11], acute respiratory distress syndrome (ARDS) [12], pneumonia, meningitis, urinary tract infection, surgery site infection, intravascular catheter-related bloodstream infection [13], pulmonary embolism, deep venous thrombosis, cerebral ischemia, hypotension (defined as need for vasopressors to keep mean arterial pressure greater than 65 mmHg), myocardial infarction [14], isolated troponin elevation without myocardial infarction, cardiac arrest, hyponatremia, hypernatremia, pressure ulcer, coagulopathy (defined as a prothrombin time international normalized ratio greater than 1.4 or platelet count less than 100,000 mm^3^) and non-convulsive status epilepticus.

In our institution only selected patients are monitored using an ICP monitor. Instead, most patients with TBI are monitored by serial clinical examination, TCD and brain computed tomography (CT), based on previously published protocols [8, 15]. In the absence of intracranial mass lesions requiring surgery, signs of intracranial hypertension on imaging or clinical examination are treated first with hyperosmolar therapies using a fixed-dose schedule of administration, optional mild hyperventilation (at a partial pressure of arterial carbon dioxide of 30–35 mmHg), and optional ventricular drainage. Administration of high-dose barbiturates or hypothermia are considered in patients with continuing edema. To estimate the impact of the different blood transfusion strategies on ICP, we therefore used a modified “integrated brain-specific treatment intensity” adapted from the Benchmark Evidence from South American Trials: Treatment of Intracranial Pressure (BEST-TRIP) trial [8], in which each strategy directed at controlling intracranial hypertension was considered as one “event”. Intensity of intracranial hypertension treatment was assessed by the need for one or more interventions directed at controlling intracranial hypertension and included the administration of hyperosmolar agents, vasopressors, the use of hyperventilation, sedation, analgesia, neuromuscular blockers, cerebral spinal fluid drainage, diuretics, hypothermia, barbiturates or decompressive craniectomy. When the ICP was monitored, it was considered elevated when greater than 20 mmHg for more than 5 min.

Neurological status was evaluated using the Glasgow Outcome Scale (GOS) [16] at hospital discharge and 6 months after hospital discharge. The neurological status evaluation was made by ambulatory consultation or home visit. When the patient was unable to come to an ambulatory consultation or a home visit was not possible, the neurological status evaluation was made by a telephone call to the patient or to a family member. The outcome assessors were blinded to the randomization assignments.

### Statistical analysis

The sample size was calculated to detect a mean difference in hemoglobin concentration between the liberal and the restrictive groups of 1.2 g/dL, assuming a mean hemoglobin concentration of 8.2 g/dL in the restrictive group and a hemoglobin concentration of 9.4 g/dL in the liberal group, with a standard deviation of 1.2 g/dL in both groups, based on previous trials [17]. Thus, enrollment of 44 patients was required for statistical power of 90% at a two-sided alpha level of 0.05. No interim analysis was planned.

Analyses were based on the modified intention-to-treat population, excluding patients whose consent was later withdrawn and excluding randomization errors. Repeated measures general linear modeling was performed to assess the overall effect of group allocation on daily mean hemoglobin during the ICU stay (interaction between the transfusion strategy and time). No imputations for missing data were performed. For continuous variables, skewness and kurtosis values were used to assess normality of the data distribution and distributional graphical methods were used. Continuous variables were analyzed as mean ± standard deviation or as median and quartiles (25th–75th) and compared by Student’s *t* test or the Wilcoxon-Mann-Whitney test, accordingly. Categorical variables were presented as absolute and relative frequencies and compared using the *χ*^2^ or Fisher’s exact test, as appropriate. Secondary outcomes were considered exploratory and no multiple test correction was implemented. All tests were two-tailed and statistical significance was assumed with a *p* value less than 0.05. The statistical analysis was performed using the SPSS software (IBM SPSS Statistics for Windows, version 24.0. Armonk, NY: IBM Corp.).

## Results

### Study patients

**Fig. 1:**
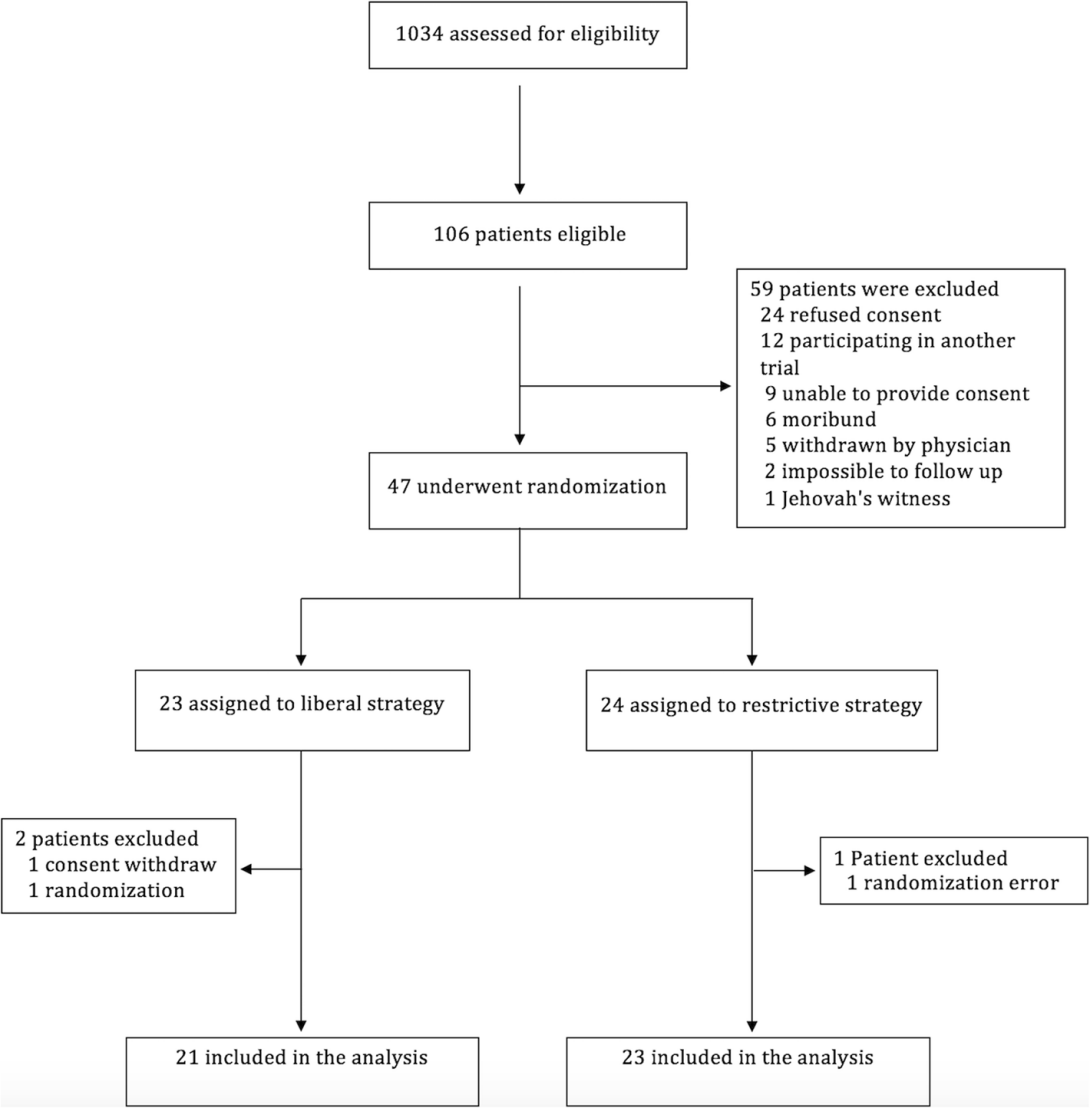
Screening and enrollment

Between August 2014 and June 2016, 1034 patients were assessed for eligibility. Of these, 106 were eligible for inclusion and 47 were randomized. After randomization, three patients were excluded, one because consent was withdrawn by the family before receiving any intervention, and two because of randomization errors (hemoglobin greater than 9 g/dL at randomization and reduced level of consciousness not attributable to TBI). Thus, 44 patients were included in the final analysis, with 21 in the liberal group and 23 in the restrictive group (Fig. 1). The study groups were statistically well-matched at baseline with respect to demographic and clinical characteristics. However, the restrictive group had more pupil alterations (13 (59%) vs. 7 (33%); *p* = 0.09); more midline deviation ≥ 5 mm on brain CT (16 (70%) vs. 10 (48%); *p* = 0.14); and more patients received blood transfusions before randomization (15 (65%) vs. 9 (43%); *p* = 0.14), compared to the liberal group (Table 1).

**Table 1:** Patient demographics

Characteristics	Total (44)	Liberal (21)	Restrictive (23)	*p* value
Age, years	35 ± 13	33 ± 11	36 ± 15	0.34
Male	40 (81)	20 (95)	20 (87)	0.61
GCS at hospital admission	4 [3–7]	4 [3–7]	5 [3–7]	0.90
Systolic blood pressure, mmHg	120 ± 24	118 ± 20	122 ± 28	0.64
Pulse rate, bpm	90 ± 25	96 ± 28	84 ± 20	0.10
Pupil alteration (one or both)	20 (46)	7 (33)	13 (59)	0.09
Patients transfused RBC before randomization	24 (55)	9 (43)	15 (65)	0.14
RBC units per patient transfused before randomization	4.4 ± 6.2	3.1 ± 5.2	5.6 ± 6.9	0.24
Patients transfused other blood products before randomization	4 (10)	0	4 (17)	0.11
Brain CT				
Compressed cisterns	35 (81)	17 (85)	18 (78)	0.70
Midline deviation ≥ 5 mm	26 (59)	10 (48)	16 (70)	0.14
Diffuse axonal lesion	14 (33)	9 (45)	5 (22)	0.10
Subarachnoid hemorrhage	22 (50)	13 (62)	9 (39)	0.13
Intracranial hematoma	35 (80)	17 (81)	18 (78)	0.82
Neurosurgical intervention				
None	11 (25)	5 (24)	6 (26)	0.86
Epidural hematoma drainage	6 (14)	4 (19)	2 (9)	0.40
Subdural hematoma drainage	5 (11)	1 (5)	4 (17)	0.35
Intracerebral hemorrhage drainage	2 (5)	1 (5)	1 (4)	1.00
Decompressive craniectomy	17 (39)	7 (33)	10 (44)	0.49
Other	5 (11)	3 (14)	2 (9)	0.66
Associated trauma lesions				
Face	31 (70)	16 (76)	15 (65)	0.43
Neck	8 (18)	6 (29)	2 (9)	0.13
Thorax	21 (48)	11 (52)	10 (44)	0.55
Abdomen	7 (16)	2 (10)	5 (22)	0.42
Pelvis and limbs	22 (50)	12 (57)	10 (44)	0.36
Spine	5 (11)	3 (14)	2 (9)	0.66
Mechanism of injury				0.66
Fall	17 (37)	7 (33)	10 (44)	
Motorcycle crash	14 (32)	6 (29)	8 (35)	
Run over	5 (11)	3 (14)	2 (9)	
Physical aggression	3 (7)	1 (5)	2 (9)	
Car/bus crash	4 (9)	3 (14)	1 (4)	
Other	1 (2)	0	0	
Pre-hospital transportation				0.638
EMS ambulance	22 (52)	12 (57)	10 (44)	
EMS helicopter	19 (43)	8 (38)	11 (48)	
Other	3 (2.4)	1 (5)	2 (9)	
Prognostic scores				
SAPS3 at ICU admission	56 ± 13	55 ± 12	57 ± 12	0.49
CRASH 14-day death risk	40 ± 18	39 ± 18	41 ± 19	0.82
CRASH 6-month unfavorable outcome risk	70 ± 19	70 ± 17	70 ± 22	0.91
Injury severity score	29 ± 9	28 ± 9	31 ± 9	0.40
IMPACT	49 (15)	47 (17)	52 (14)	0.29
Injury-to-randomization time, h	71 ± 38	75 ± 41	65 ± 34	0.35

Values are expressed as the mean ± standard deviation, median [25th–75th percentiles] or number (percentage)

*GCS* Glasgow Come Scale, *RBC* red blood cells, *EMS* Emergency Medical Service, *CT* computer tomograph, *SAPS* Simplified Acute Physiology Score, *ICU*, Intensive care unit, *CRASH* Corticoid Randomization After Significant Head injury Score, *IMPACT* International Mission for Prognosis And Clinical Trial Score

### Primary outcome

**Fig. 2:**
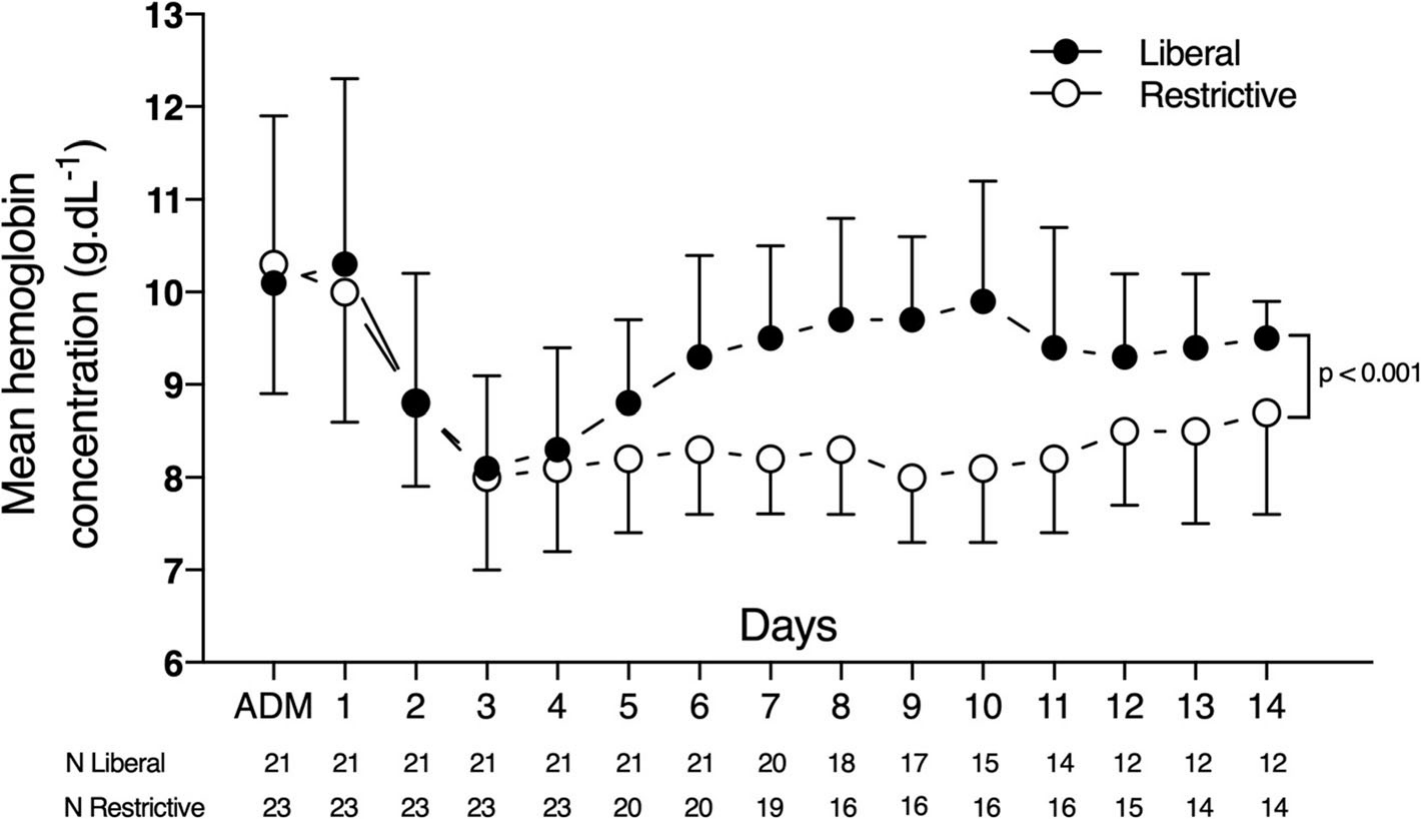
Mean daily hemoglobin concentrations in the liberal and restrictive strategy groups during the first 14 days after intensive care unit (ICU) admission (ADM)

The mean hemoglobin concentration during the first 14 days after hospital admission was 9.3 ± 1.3 g/dL in the liberal group and 8.4 ± 1.0 g/dL in the restrictive group (*p* < 0.01), giving a mean difference of 0.9 ± 0.2 g/dL (Table 2). This difference gradually increased after the fourth day, to a peak on the tenth day, when the difference was 1.8 ± 0.4 g/dL (CI 95% 1.0–2.6, *p* < 0.01) (Fig. 2). There was a significant interaction between the transfusion strategy and time regarding the daily mean hemoglobin during the ICU stay from day 1 to day 14 (*p* = 0.018). The post-hoc analysis revealed that the difference between the groups in daily mean hemoglobin was statistically significant from day 5 up to day 14.

**Table 2:** Outcomes

Outcomes	Total (44)	Liberal (21)	Restrictive (23)	*p* value
Hemoglobin concentration, g/dL				
14-day, g/dL	8.8 ± 1.2	9.3 ± 1.3	8.4 ± 1.0	< 0.01
Hospital admission	12.3 ± 2.0	12.0 ± 2.3	12.5 ± 1.8	0.53
ICU admission	10.2 ± 1.4	10.1 ± 1.2	10.3 ± 1.6	0.55
Pre-randomization	8.1 ± 0.8	7.9 ± 0.6	8.2 ± 1.0	0.34
Patients transfused	34 (77)	21 (100)	13 (57)	< 0.01
Total transfused RBC units	101	66	35	0.02
RBC units per patient	2.3 ± 1.8	3.1 ± 1.6	1.5 ± 1.7	< 0.01
Other blood products	0	0	0	0
Mortality				
ICU death	8 (18)	1 (5)	7 (30)	0.05
In-hospital death	8 (18)	1 (5)	7 (30)	0.05
Discharge functional outcome (GOS)				
Ordinal				0.06
1 – Death	8 (18)	1 (5)	7 (30)	
2 – Vegetative state	7 (16)	3 (14)	4 (17)	
3 – Severe disability	6 (14)	4 (19)	2 (9)	
4 – Moderate disability	16 (36)	9 (43)	7 (30)	
5 – Good recovery	7 (16)	4 (19)	3 (13)	
Dichotomous				0.22
Unfavorable (GOS–3)	21 (48)	8 (38)	13 (57)	
Favorable (GOS 4–5)	23 (52)	13 (62)	10 (44)	
Length of stay, days				
ICU	17 [10–28]	21 [9–30]	16 [13–18]	0.35
Hospital	39 [23–66]	35 [21–63]	42 [23–76]	0.59
Functional outcome (GOS) at 6 months				
Ordinal				0.06
1 – Death	9 (21)	2 (10)	7 (30)	
2 – Vegetative state	5 (11)	2 (10)	3 (13)	
3 – Severe disability	7 (16)	4 (19)	3 (13)	
4 – Moderate disability	8 (18)	3 (14)	5 (22)	
5 – Good recovery	15 (34)	10 (48)	5 (22)	
Dichotomous				0.20
Unfavorable (GOS–3)	21 (48)	8 (38)	13 (57)	
Favorable (GOS 4–5)	23 (52)	13 (62)	10 (44)	

Values are expressed as the mean ± standard deviation, median [25th–75th percentiles] or number (percentage)

*ICU* intensive care unit, *RBC* red blood cell, *GOS* Glasgow Outcome Scale

### Secondary outcomes

#### Transfusion

Adherence to the protocol was good throughout the study with a few exceptions. In two patients who were assigned to the liberal group, the strategy was not maintained for the full 14 days or until ICU discharge. One patient was managed according to the restrictive strategy after developing ARDS and risk of developing transfusion-associated circulatory overload (TACO). In the other patient, the attending physician refused to give a transfusion one day after randomization, despite a hemoglobin concentration lower than 9 g/dL.

After randomization, the median lowest daily hemoglobin concentrations were 7.9 (7.3–8.7) g/dL and 6.8 (6.6–7.9) g/dL in the liberal and restrictive groups, respectively (*p* = 0.02). All 21 patients in the liberal group were transfused, compared with 13 (57%) patients in the restrictive group, with a mean of 3.1 ± 1.6 vs. 1.5 ± 1.7 units per patient, respectively (*p* < 0.01) (Table 2).

#### Complications

There were no differences in the numbers of complications, individually or combined, in the liberal and restrictive groups: the total numbers of complications per patient were 4.2 ± 1.8 vs. 4.1 ± 1.7, respectively (*p* = 0.86) (Additional file 1: Table S1).

The interventions aiming at controlling intracranial hypertension are shown in Additional file 1: Table S2. Although hypertonic saline was more frequently administered to the liberal group (10/23, 32% vs. 4/21, 17%; *p* = 0.03), there was no difference in the total number of interventions between the liberal and restrictive groups.

#### Transcranial Doppler ultrasound findings

The mean values of the TCD analysis from the first 14 days after ICU admission are shown in Additional file 1: Table S3. The restrictive group had higher flow velocities in all studied cerebral arteries compared to the liberal group. There was negative correlation (*r* = − 0.265, *p* < 0.01) between the hemoglobin concentration and middle cerebral artery flow velocity. The incidence of post-traumatic vasospasm was significantly lower in the liberal group (4/21, 3% vs. 15/23, 65%; *p* < 0.01). There were no statistically significant differences in pulsatility indexes (PI) between groups.

#### Neurological status and mortality

**Fig. 3:**
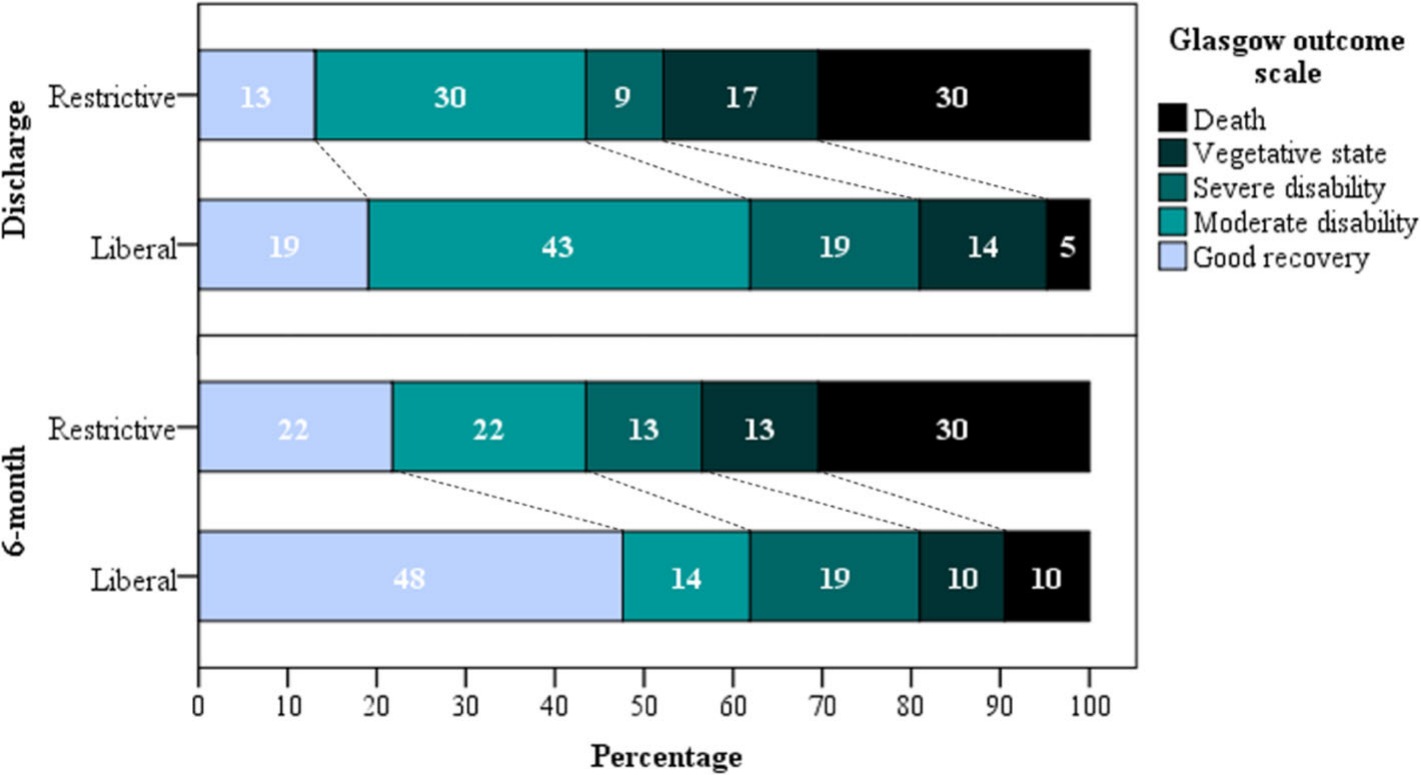
Neurological status at hospital discharge and at 6 months after hospital discharge as measured by the Glasgow Outcome Scale

Survival data were available for all patients at ICU discharge, at hospital discharge and at 6 months after hospital discharge. Eight patients died during the hospital stay, all of them in the ICU (Table 2). Hospital mortality was lower in the liberal group (*n* = 1 patient (5%) vs. *n* = 7 patients (30%), respectively; *p* = 0.048). There was no significant difference between groups in GOS outcomes at hospital discharge. At 6 months, nine patients had died, two in the liberal group and seven in the restrictive group. The liberal group tended to have a better neurological status, as evaluated by the GOS (*p* = 0.06). Furthermore, among the living patients, 62% vs. 44% had a favorable GOS in the liberal and restrictive groups, respectively (Fig. 3, Table 2).

## Discussion

There was a significant difference in hemoglobin concentrations and use of RBC units between the two transfusion strategies. The restrictive group had lower hemoglobinconcentrations, and received fewer RBC transfusions; the number of complications was not different between groups. Furthermore, hospital mortality was higher in the restrictive group and neurological status at 6 months favored the liberal group.

Several studies have evaluated different transfusion thresholds in diverse critically ill populations; however, only two randomized clinical trials have included patients with TBI [18]. Both trials compared hemoglobin transfusion thresholds of 7 g/dL and 10 g/dL, and concluded that the restrictive threshold was safe. A liberal threshold of 9 g/dL was chosen because (1) this hemoglobin concentration has been associated with cerebral hypoxia in experimental studies [3, 19, 20] and (2) it is the most widely used hemoglobin concentration threshold in international surveys evaluating patients with TBI [6, 7].

Adherence to the protocol was good throughout the study with only two protocol violations, both in the liberal group. In fact, the greatest challenge to protocol adherence was the blood transfusion trigger targeting a hemoglobin concentration greater than 9 g/dL in the liberal group, especially in patients with no evidence of intracranial hypertension. This trigger was often seen by the attending physician as potentially harmful, given the guideline recommendations to target a hemoglobin concentration of 7 to 9 g/dL [21].

The difference in the mean hemoglobin concentration between the restrictive and liberal groups was 0.9 ± 0.2 g/dL. Although this difference was statistically significant, it may not be clinically relevant. However, this number represents the mean hemoglobin concentration over the full 14 day-period after hospital admission, and the day-by-day differences were considerably higher.

Our results differ from those of Robertson et al. [5] in which maintaining a hemoglobin concentration of at least 10 g/dL did not result in improved neurological outcome at 6 months. However, these authors included a different TBI population and did not use hemoglobin concentration as an inclusion criterion. As a result, both groups had hemoglobin average concentrations greater than 9 g/dL at all reported time points. This hemoglobin concentration is higher than the value that has been associated with cerebral hypoxia in experimental studies [3, 19, 20]. In the present study, by including only patients with TBI with a hemoglobin concentration less than 9 g/dL, we created a difference between the groups, using a real restrictive transfusion strategy in the control group. The restrictive group had higher mean flow velocities in all studied cerebral arteries. These results are expected, because hemoglobin concentrations are directly correlated with flow velocities on TCD [22]. Furthermore, the restrictive group had a higher incidence of post-traumatic vasospasm than the liberal group. Post-traumatic vasospasm has been reported in up to 61% of patients with severe TBI and is associated with cerebral ischemia, longer ICU and hospital stay and worse neurological outcomes [9, 10]. However, we do not have confirmatory data that could be obtained by angiography.

Hospital mortality was significantly lower in the liberal group than in the restrictive group, and neurological outcome at 6 months also tended to favor the liberal group. These observations may be related to impaired cerebral oxygenation in patients in the restrictive group [3]. However, cerebral oxygenation was not measured in our study, so we were unable to confirm this mechanism. Another hypothesis is that the worse outcomes in the restrictive group may have been related to the higher incidence of cerebral post-traumatic vasospasm as detected by serial transcranial Doppler.

The study groups were statistically well-matched at baseline with respect to demographic and clinical characteristics. However, the restrictive group had more pupil alterations, more midline deviations ≥ 5 mm on brain CT, and more patients received blood transfusions before randomization compared to the liberal group. Although not statistically significant, it might suggest that the restrictive group would be more severely affected than the liberal group, and could explain part of the reported secondary outcomes.

Overall, the intensity of intracranial hypertension treatment was not different in the two groups, although there was more hypertonic saline use in the liberal group. It could mean the liberal group were better treated for intracranial hypertension than the restrictive group, however, there was no evidence that the liberal group had more events of uncontrolled intracranial hypertension when analyzing data from TCD or clinical outcomes.

There were no statistically significant differences in the occurrence of adverse events in the two groups. The incidence of clinically evident deep venous thrombosis was higher in the liberal group (3 vs. 0 events), similar to the results of Robertson et al. [5], but this difference was not statistically significant.

Several limitations should be noted. First, this was a pilot trial, aimed at evaluating the feasibility of a randomized clinical trial comparing liberal and restrictive blood transfusion strategies in patients with moderate and severe TBI. Therefore, the secondary outcome analysis should be considered exploratory. Nevertheless, our results are in line with previous experimental, physiological and observational data and point toward superiority of the liberal transfusion strategy [3, 19, 20]. Second, the sample size was small and may have been underpowered to detect small differences between groups. Third, the patients were evaluated at hospital discharge and 6 months after hospital discharge, instead of at fixed time points. However, hospital discharge was at the discretion of the assistant team and not influenced by the investigators, and the length of hospital stay was not different between groups. Fourth, this was a single-center trial and recruitment was slow, taking 2 years to include 44 patients. Our main difficulty was obtaining written informed consent. Thirty-five patients refused or were unable to provide consent, which prolonged the trial and may have delayed the intervention. A strategy of delayed consent may increase recruitment in future trials. Fifth, ICP was often not monitored. However, this was a pragmatic trial and ICP monitoring is not part of routine practice in our ICU.

## Conclusions

The trial reached feasibility criteria. The restrictive group had lower hemoglobin concentrations and received fewer RBC transfusions. Hospital mortality was lower and neurological status at 6 months favored the liberal group. Given the observed difference in outcome, a phase-II study is warranted.

## Additional file

Additional file 1: **Table S1.** Adverse events. **Table S2.** Interventions for intracranial hypertension control during the ICU stay. **Table S3.** Transcranial Doppler. **Table S4.** Patients’ comorbidities. (DOCX 25 kb)

## Abbreviations

ARDS: Acute respiratory distress syndrome
BEST-TRIP: Benchmark Evidence From South American Trials Treatment Of Intracranial Pressure
Cappesq: Comissão de Ética para Análise de Projetos de Pesquisa
GCS: Glasgow Coma Scale
GOS: Glasgow Outcome Scale
ICP: Intracranial pressure
ICU: Intensive care unit
RBC: Red blood cells
SPSS: Statistical Package for The Social Sciences
TACO: Transfusion-associated circulatory overload
TBI: Traumatic brain injury
TCD: Transcranial Doppler
TRAHT: Transfusion Requirements After Head Trauma

## References

[1] Vincent JL, Baron JF, Reinhart K, Gattinoni L, Thijs L, Webb A, Meier-Hellmann A, Nollet G, Peres-Bota D, Investigators ABC (2002). Anemia and blood transfusion in critically ill patients. JAMA.

[2] Salim A, Hadjizacharia P, DuBose J, Brown C, Inaba K, Chan L, Margulies DR (2008). Role of anemia in traumatic brain injury. J Am Coll Surg.

[3] Oddo M, Levine JM, Kumar M, Iglesias K, Frangos S, Maloney-Wilensky E, Le Roux PD (2012). Anemia and brain oxygen after severe traumatic brain injury. Intensive Care Med.

[4] McIntyre LA, Fergusson DA, Hutchison JS, Pagliarello G, Marshall JC, Yetisir E, Hare GM, Hebert PC (2006). Effect of a liberal versus restrictive transfusion strategy on mortality in patients with moderate to severe head injury. Neurocrit Care.

[5] Robertson CS, Hannay HJ, Yamal JM, Gopinath S, Goodman JC, Tilley BC, Epo Severe TBITI, Baldwin A, Rivera Lara L, Saucedo-Crespo H (2014). Effect of erythropoietin and transfusion threshold on neurological recovery after traumatic brain injury: a randomized clinical trial. JAMA.

[6] Sena MJ, Rivers RM, Muizelaar JP, Battistella FD, Utter GH (2009). Transfusion practices for acute traumatic brain injury: a survey of physicians at US trauma centers. Intensive Care Med.

[7] Badenes R, Oddo M, Suarez JI, Antonelli M, Lipman J, Citerio G, Taccone FS (2017). Hemoglobin concentrations and RBC transfusion thresholds in patients with acute brain injury: an international survey. Crit Care.

[8] Chesnut RM, Temkin N, Carney N, Dikmen S, Rondina C, Videtta W, Petroni G, Lujan S, Pridgeon J, Barber J (2012). A trial of intracranial-pressure monitoring in traumatic brain injury. N Engl J Med.

[9] Perrein A, Petry L, Reis A, Baumann A, Mertes P, Audibert G (2015). Cerebral vasospasm after traumatic brain injury: an update. Minerva Anestesiol.

[10] Oertel M, Boscardin WJ, Obrist WD, Glenn TC, McArthur DL, Gravori T, Lee JH, Martin NA (2005). Posttraumatic vasospasm: the epidemiology, severity, and time course of an underestimated phenomenon: a prospective study performed in 299 patients. J Neurosurg.

[11] Dellinger RP, Levy MM, Rhodes A, Annane D, Gerlach H, Opal SM, Sevransky JE, Sprung CL, Douglas IS, Jaeschke R (2013). Surviving Sepsis Campaign: international guidelines for management of severe sepsis and septic shock, 2012. Intensive Care Med.

[12] Force ADT, Ranieri VM, Rubenfeld GD, Thompson BT, Ferguson ND, Caldwell E, Fan E, Camporota L, Slutsky AS (2012). Acute respiratory distress syndrome: the Berlin Definition. JAMA.

[13] Mermel LA, Allon M, Bouza E, Craven DE, Flynn P, O'Grady NP, Raad II, Rijnders BJ, Sherertz RJ, Warren DK (2009). Clinical practice guidelines for the diagnosis and management of intravascular catheter-related infection: 2009 update by the Infectious Diseases Society of America. Clin Infect Dis.

[14] Thygesen K, Alpert JS, Jaffe AS, Simoons ML, Chaitman BR, White HD, Katus HA, Lindahl B, Morrow DA, Joint ESC/ACCF/AHA/WHF Task Force for the universal definition of myocardial infarction (2012). Third universal definition of myocardial infarction. Circulation.

[15] Ferreira CB, Bassi E, Lucena L, Carreta H, Miranda LC, Tierno PF, Amorim RL, Zampieri FG, Malbouisson LM (2015). Measurement of intracranial pressure and short-term outcomes of patients with traumatic brain injury: a propensity-matched analysis. Rev Bras Ter Intensiva.

[16] Teasdale GM, Pettigrew LE, Wilson JT, Murray G, Jennett B (1998). Analyzing outcome of treatment of severe head injury: a review and update on advancing the use of the Glasgow Outcome Scale. J Neurotrauma.

[17] McIntyre L, Hebert PC, Wells G, Fergusson D, Marshall J, Yetisir E, Blajchman MJ, Canadian Critical Care Trials G (2004). Is a restrictive transfusion strategy safe for resuscitated and critically ill trauma patients?. J Trauma.

[18] Boutin A, Chasse M, Shemilt M, Lauzier F, Moore L, Zarychanski R, Griesdale D, Desjardins P, Lacroix J, Fergusson D (2016). Red blood cell transfusion in patients with traumatic brain injury: a systematic review and Meta-analysis. Transfus Med Rev.

[19] Smith MJ, Stiefel MF, Magge S, Frangos S, Bloom S, Gracias V, Le Roux PD (2005). Packed red blood cell transfusion increases local cerebral oxygenation. Crit Care Med.

[20] Zygun DA, Nortje J, Hutchinson PJ, Timofeev I, Menon DK, Gupta AK (2009). The effect of red blood cell transfusion on cerebral oxygenation and metabolism after severe traumatic brain injury. Crit Care Med.

[21] Rossaint R, Bouillon B, Cerny V, Coats TJ, Duranteau J, Fernandez-Mondejar E, Filipescu D, Hunt BJ, Komadina R, Nardi G (2016). The European guideline on management of major bleeding and coagulopathy following trauma: fourth edition. Crit Care.

[22] Lagunju I, Sodeinde O, Brown B, Akinbami F, Adedokun B (2014). Transcranial Doppler ultrasonography in children with sickle cell anemia: clinical and laboratory correlates for elevated blood flow velocities. J Clin Ultrasound.

