# Supplementary material for: Transfusion requirements after head trauma: a randomized feasibility controlled trial
Source: Crit Care. 2019 Mar 12;23:89. doi: 10.1186/s13054-018-2273-9 (PMC6419414; doi:10.1186/s13054-018-2273-9)
Supplement: Supplementary file 1 — Table S1. Adverse events. Table S2. Interventions for intracranial hypertension control during the ICU stay. Table S3. Transcranial Doppler. Table S4. Patients’ comorbidities. (DOCX 25 kb) [file 13054_2018_2273_MOESM1_ESM.docx]

| **TABLE S1. Adverse events** | | | | | |
| --- | --- | --- | --- | --- | --- |
|  | **Total (44)** | | **Liberal (21)** | **Restrictive (23)** | **p-value** |
| **Hypotension** | 38 (86) | 18 (86) | | 20 (87) | 1.00 |
| **Acute myocardial infarction** | 0 (0) | 0 (0) | | 0 (0) | - |
| **Septic shock** | 16 (36) | 5 (24) | | 11 (48) | 0.10 |
| **Pneumonia** | 14 (32) | 5 (24) | | 9 (39) | 0.28 |
| **Bloodstream infection** | 5 (11) | 2 (10) | | 3 (13) | 1.00 |
| **Urinary tract infection** | 3 (7) | 3 (14) | | 0 (0) | 0.10 |
| **Meningitis** | 2 (5) | 1 (5) | | 1 (4) | 1.00 |
| **Surgical site infection** | 1 (3) | 1 (6) | | 0 (0) | 1.00 |
| **ARDS** | 2 (6) | 1 (6) | | 1 (6) | 1.00 |
| **Pulmonary embolism** | 2 (5) | 1 (5) | | 1 (4) | 0.48 |
| **Stroke (ischemic or hemorrhagic)** | 2 (5) | 2 (10) | | 0 (0) | 0.23 |
| **Non-convulsive epileptic status** | 1 (2) | 0 (0) | | 1 (4) | 1.00 |
| **Hypernatremia** | 33 (75) | 17 (81) | | 16 (70) | 0.38 |
| **Hyponatremia** | 14 (32) | 9 (43) | | 5 (22) | 0.13 |
| **Coagulopathy** | 22 (50) | 10 (48) | | 12 (52) | 0.76 |
| **Pressure ulcers** | 3 (7) | 1 (5) | | 2 (9) | 1.00 |
| **Deep venous thrombosis** | 3 (7) | 3 (14) | | 0 (0) | 0.10 |
| **Tracheostomy** | 21 (48) | 10 (48) | | 11 (48) | 0.10 |
| **Complications per patient** | 4 ± 2 | 4 ± 2 | | 4 ± 2 | 0.66 |
| **Values are expressed as the mean ± standard deviation, median [25th-75th percentiles] or number (percentage). ARDS: Acute Respiratory Distress Syndrome** | | | | | |

| **TABLE S2. Interventions for intracranial hypertension control during ICU stay** | | | | |
| --- | --- | --- | --- | --- |
|  | **Total (44)** | **Liberal (21)** | **Restrictive (23)** | **p-value** |
| **One or more interventions** | 43 (98) | 20 (95) | 23 (100) | - |
| Analgesia | 42 (96) | 19 (91) | 23 (100) | 0.22 |
| Sedation | 42 (96) | 19 (91) | 23 (100) | 0.22 |
| Vasopressors | 36 (82) | 17 (81) | 19 (83) | 1.00 |
| Hypertonic saline | 14 (32) | 10 (48) | 4 (17) | 0.03 |
| Neuromuscular paralysis | 7 (16) | 4 (19) | 3 (13) | 0.69 |
| Hyperventilation | 4 (9) | 0 (0) | 4 (17) | 0.11 |
| Mannitol | 3 (7) | 2 (10) | 1 (4) | 0.60 |
| Hypothermia | 3 (7) | 1 (5) | 2 (9) | 1.00 |
| Barbiturates | 3 (7) | 2 (10) | 1 (4) | 0.6 |
| CSF drainage | 2 (5) | 1 (5) | 1 (4) | 1.00 |
| **Total number of interventions per patient (mean ± SD)** | 17 ± 11 | 18 ± 13 | 16 ± 9 | 0.50 |
| **Values are expressed as the mean ± standard deviation, median [25th-75th percentiles] or number (percentage). ICU: Intensive Care Unit; CSF: Cerebral Spinal Fluid.** | | | | |

| **TABLE S3. Transcranial Doppler** | | | | |
| --- | --- | --- | --- | --- |
|  | **Total (44)** | **Liberal (21)** | **Restrictive (23)** | **p-value** |
| **Flow Velocity** |  |  |  |  |
| Right internal carotid artery, cm/s | 34.5 ± 7.6 | 33.1 ± 7.3 | 35.7 ± 7.6 | <0.01 |
| Left internal carotid artery, cm/s | 34.9 ± 8.3 | 33.2 ± 7.9 | 36.2 ± 8.4 | <0.01 |
| Right middle cerebral artery, cm/s | 79.1 ± 24.2 | 70.2 ± 21.3 | 86.9 ± 23.9 | <0.01 |
| Left middle cerebral artery, cm/s | 78.0 ± 22.4 | 72.6 ± 22.5 | 82.8 ± 21.3 | <0.01 |
| **Pulsatility Index** |  |  |  |  |
| Right | 1.0 ± 0.2 | 1.0 ± 0.2 | 1.0 ± 0.2 | 0.55 |
| Left | 1.0 ± 0.3 | 1.0 ± 0.3 | 1.0 ± 0.2 | 0.55 |
| Main affected side | 1.0 ± 0.2 | 1.0 ± 0.2 | 1.0 ± 0.2 | 0.59 |
| Maximum | 1.1 ± 0.3 | 1.1 ± 0.3 | 1.0 ± 0.2 | 0.25 |
| **Lindegaard Index** |  |  |  |  |
| Right | 2.4 ± 0.8 | 2.2 ± 0.8 | 2.5 ± 0.8 | <0.01 |
| Left | 2.3 ± 0.8 | 2.3 ± 0.9 | 2.4 ± 0.7 | 0.21 |
| Main affected side | 2.4 ± 0.8 | 2.2 ± 0.9 | 2.4 ± 0.7 | 0.02 |
| Maximum | 2.6 ± 0.8 | 2.5 ± 0.9 | 2.7 ± 0.7 | 0.04 |
| **Vasospasm** |  |  |  |  |
| Main affected side | 16 (5) | 4 (3) | 12 (7) | 0.10 |
| Any side | 19 (6) | 4 (3) | 15 (9) | 0.03 |
| Classification |  |  |  | 0.65 |
| Mild | 13 (68) | 3 (75) | 10 (67) |  |
| Moderate | 5 (26) | 1 (25) | 4 (27) |  |
| Severe | 1 (5) | 0 (0) | 1 (6) |  |
| **The values represent the mean values over the first 14 days after hospital admission. Values are expressed as the mean ± standard deviation, median [25th-75th percentiles] or number (percentage).** | | | | |

| **TABLE S4. Patients comorbidities** | | | | | |
| --- | --- | --- | --- | --- | --- |
| **Characteristics** | **Total (44)** | | **Liberal (21)** | **Restrictive (23)** | **p-value** |
| **Hypertension** | 2 (4) | 1 (5) | | 1 (4) | 0.95 |
| **Diabetes Mellitus** | 0 | 0 | | 0 | 0 |
| **Dyslipidemia** | 1 (2) | 0 | | 1 (4) | 0.29 |
| **Smoking** | 2 (4) | 1 (5) | | 1 (4) | 1 |
| **Chronic Kidney Disease** | 0 | 0 | | 0 | 0 |
| **COPD** | 0 | 0 | | 0 | 0 |
| **Heart Failure** | 0 | 0 | | 0 | 0 |
| **Coronary Artery Disease** | 1 (2) | 0 | | 1 (4) | 0.29 |
| **Cancer** | 0 | 0 | | 0 | 0 |
| **Other** | 3 (7) | 0 | | 3 (13) | 0.45 |
| **Values are expressed as the mean ± standard deviation, median [25th-75th percentiles] or number (percentage).** | | | | | |
